# Additional data on stability of black carrot extract-loaded liposomes during storage

**DOI:** 10.1016/j.dib.2018.10.011

**Published:** 2018-10-09

**Authors:** Burcu Guldiken, Monika Gibis, Dilek Boyacioglu, Esra Capanoglu, Jochen Weiss

**Affiliations:** a Department of Food Engineering, Faculty of Chemical and Metallurgical Engineering, Istanbul Technical University, 34469 Maslak, Istanbul, Turkey; b Department of Food Physics and Meat Science, Institute of Food Science and Biotechnology, University of Hohenheim, Garbenstrasse 21/25, 70599 Stuttgart, Germany

## Abstract

The stability of black carrot extract-loaded liposomes (0.1% and 0.2% extract) was presented as additional data related to the research article entitled “Physical and Chemical Stability of Anthocyanin-rich Black Carrot Extract Loaded Liposomes During Storage” (Guldiken et al., 2018) [1]. This article provides further information and data on physical and chemical stability considering lower extract concentrations during storage of extract-loaded liposomes. The lower the amount of extract and higher the lecithin concentration the faster the loss of the red color is visible.

**Specifications table**

**Table:** 

Subject area	*Food Science and Technology*
More specific subject area	*Liposome encapsulation*
Type of data	*Figure*
How data was acquired	*UV/VIS-spectrophotometer, chroma meter*
Data format	*Analyzed*
Experimental factors	*Black carrot extract solutions were prepared by dissolving the liposomes in an acetate buffer (pH 3.5, 250 mM) as three independent replicates for each sample (0.1% and 0.2% w/w)*
Experimental features	*Analysis of extract concentration, total phenolic content, total antioxidant capacity*
Data source location	*Istanbul Technical University*
Data accessibility	*Data are available within this article*
Related research article	Guldiken, B., et al., *Physical and Chemical Stability of Anthocyanin-rich Black Carrot Extract Loaded Liposomes During Storage.* Food Research International, 2018. **108**: p. 491–497 [1]

**Value of the data**

• The data can be used to show the biochemical stability of 0.1% and 0.2% black carrot extract-loaded liposomes.
• The data present color attributes of stored extract loaded liposomes and can be used to determine lecithin content.
• The figures provide visual observations of extract loaded liposomes during storage.

## 1 Data

**Fig. 1:**
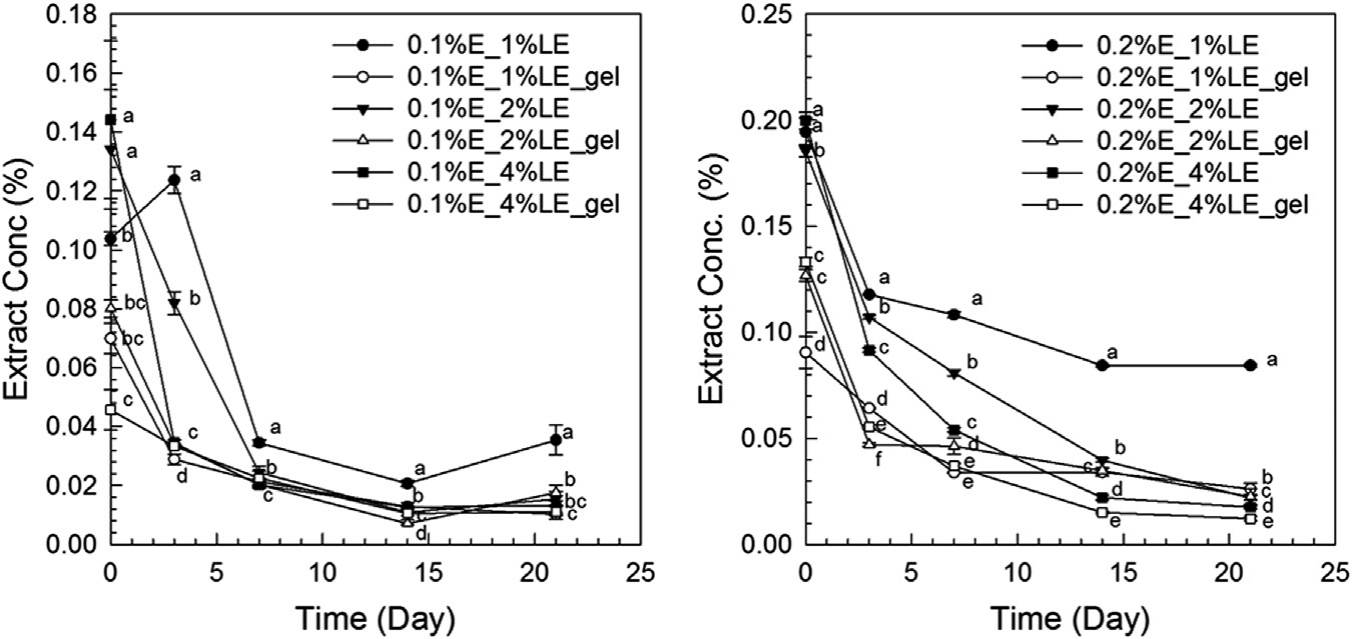
Degradation of anthocyanin in liposomes with 0.1% and 0.2% extract during storage period (without and with gel-filtration [_gel]). Data represent mean ± standard deviation of three replicates from each sample. Different lower-case letters at the same storage time represent statistically significant differences (*p* < 0.05).

**Fig. 2:**
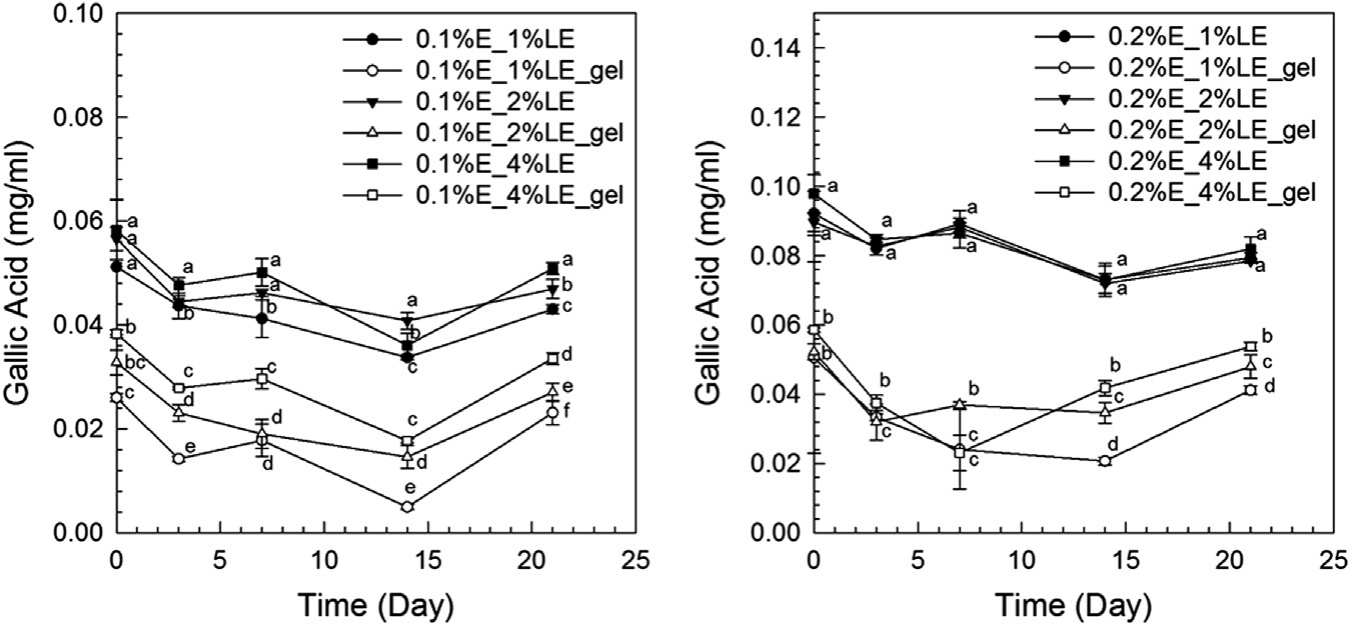
The content of total phenolic compounds in liposomes with 0.1% and 0.2% extract during storage (without and with gel-filtration [_gel]). Data represent mean ± standard deviation of three replicates from each sample. Different lower-case letters at the same storage time represent statistically significant differences (*p* < 0.05).

**Fig. 3:**
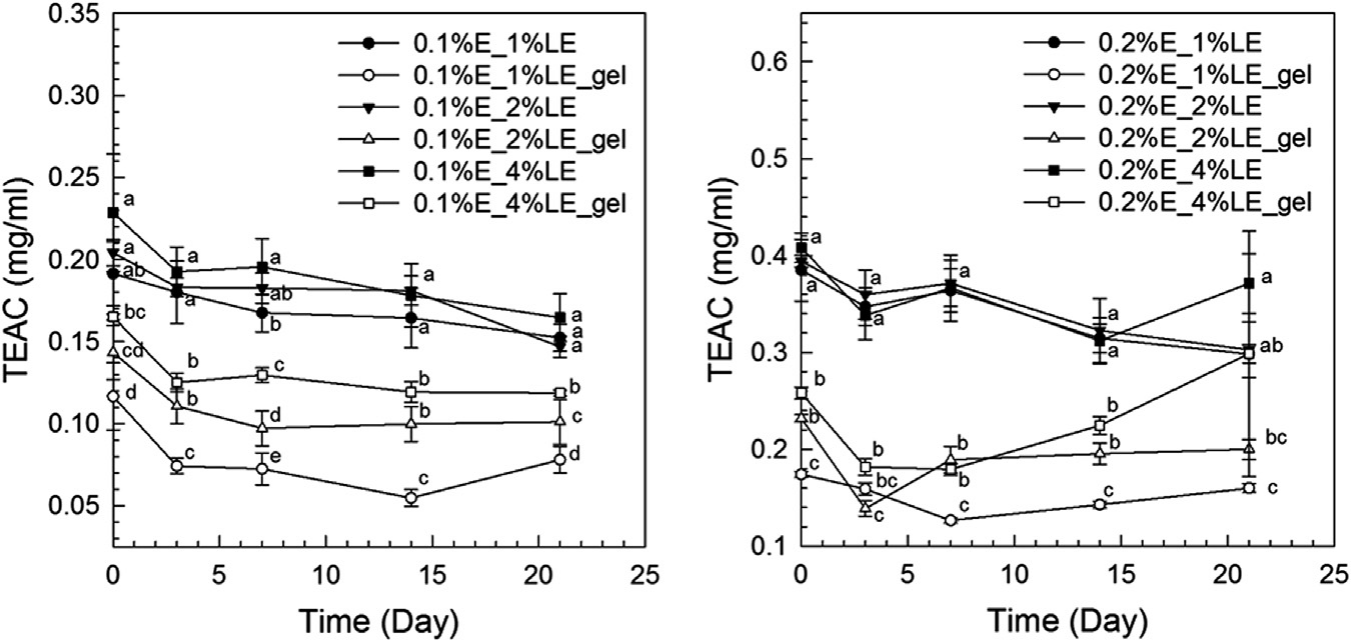
Antioxidant capacity of liposomes with 0.1% and 0.2% extract during storage (without and with gel-filtration [_gel]). Data represent average values ± standard deviation of three replicates from each sample. Different lower-case letters at the same storage time represent statistically significant differences (*p* < 0.05).

**Fig. 4:**
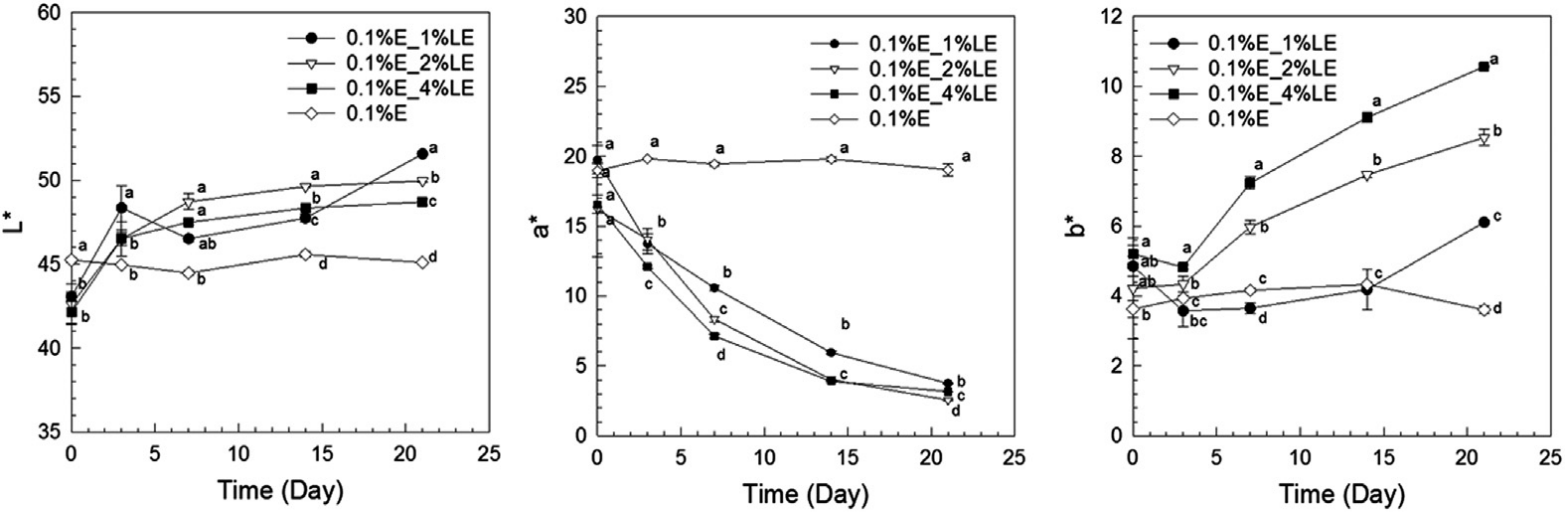
Color attributes of 0.1% extract and liposomes with 0.1% extract. Data represent mean ± standard deviation of three replicates from each sample. Different lower-case letters at the same storage time represent statistically significant differences (*p* < 0.05).

**Fig. 5:**
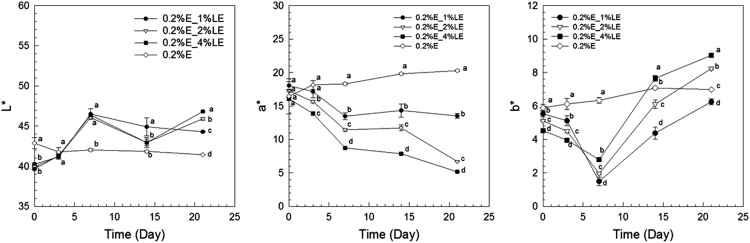
Color attributes of 0.2% extract and liposomes with 0.2% extract. Data represent mean ± standard deviation of three replicates from each sample. Different lower-case letters at the same storage time represent statistically significant differences (*p* < 0.05).

**Fig. 6:**
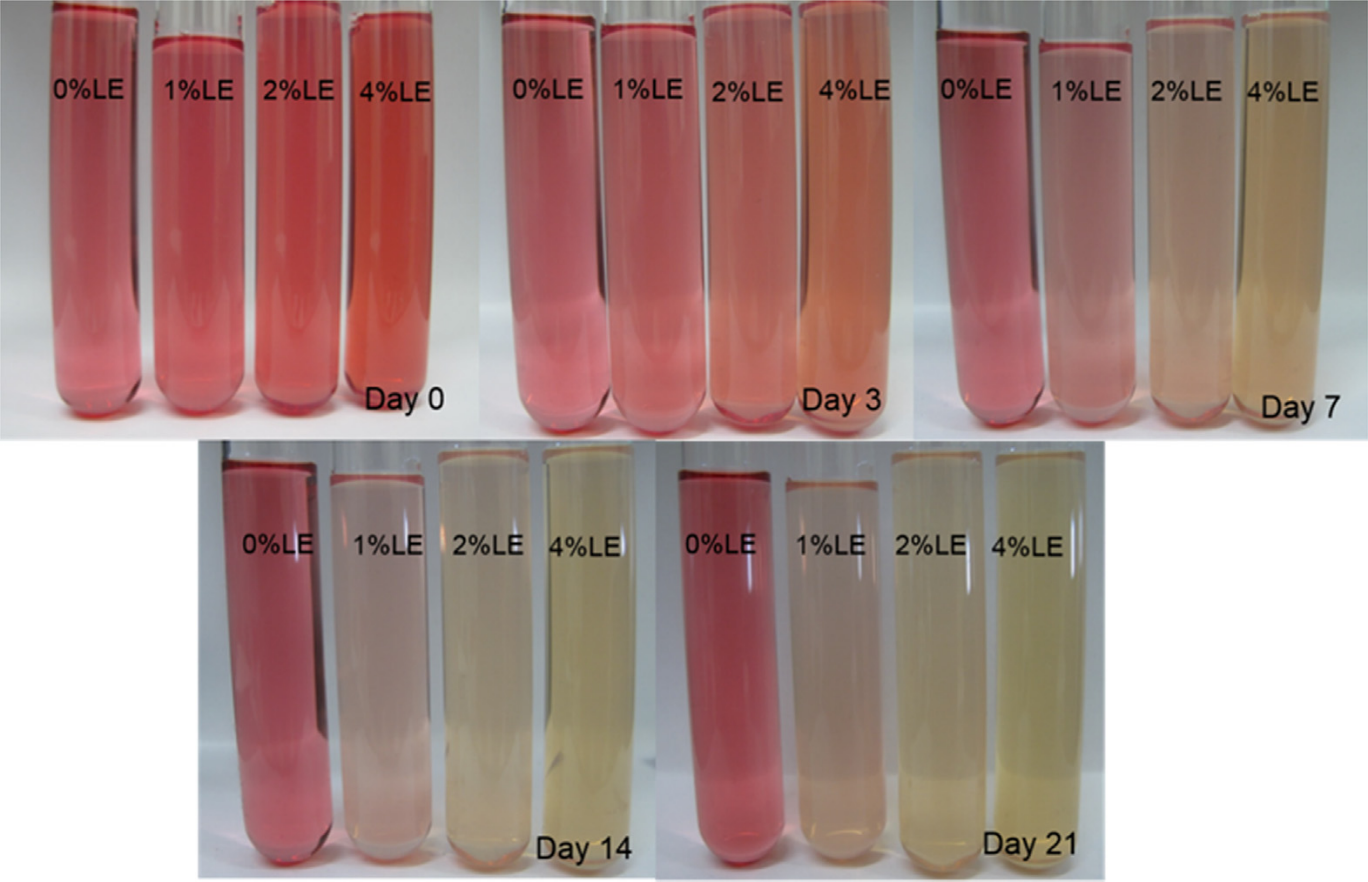
Visual observations of liposomes with 0.1% extract during storage period.

**Fig. 7:**
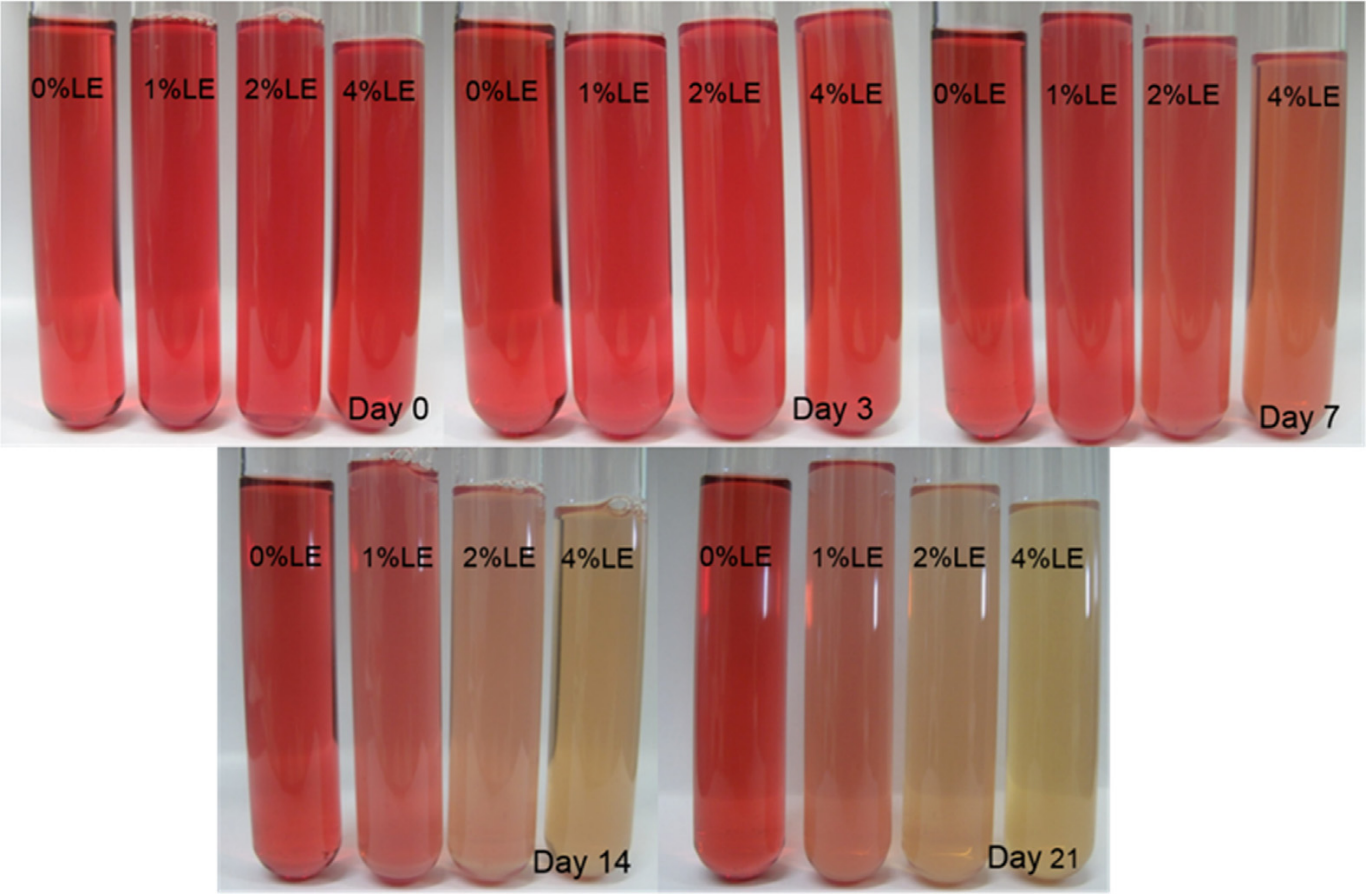
Visual observations of liposomes with 0.2% extract during storage period.

The anthocyanin amounts in samples of extract-loaded liposomes were measured. The degradation of extract using concentrations of 0.1% and 0.2% black carrot extract incorporated in liposomes is present in Fig. 1. The highest extract concentrations were found in liposomes produced with 1% lecithin for all levels of black carrot extract during 21 days of storage. The total phenolic content of extract-loaded liposomes was analyzed during storage (Fig. 2). The lower concentrations of extract (0.1%) incorporated in liposomes showed a degradation on total phenolic compounds in terms of gallic acid according to their lecithin content; however, the difference of contents of total phenolic compounds using extract encapsulated in liposomes (0.2% extract) with different lecithin contents was not significant without gel filtration. In addition, antioxidant capacity of the extract encapsulated in liposomes was evaluated during storage (Fig. 3). After storage, the highest antioxidant capacity was found in samples with the lowest lecithin content (1%) among liposomes containing 0.2% extract. Liposomal solutions contained both entrapped and non-entrapped bioactive compounds during storage. For this reason, the non-entrapped extract was removed via gel-filtration. However, the degradation trend of bioactive compounds such as extract concentration (Fig. 1) was comparable for liposomes without and with gel-filtration however of course, the levels were lower. The levels of total phenolic compounds (Fig. 2) and antioxidant capacity (Fig. 3) only slightly changed during storage since the phenolic groups did not degraded. The color attributes of extract-loaded liposomes were changed during storage regarding extract and lecithin concentration. Fig. 4, Fig. 5 present color attributes of liposomes with 0.1% and 0.2% extract, respectively. In these figures, the highest increase in yellowness and decrease in redness were found in samples containing highest lecithin content (4%) with all extract concentrations. The visual observations of liposomes containing 0.1% and 0.2% extract are given in Fig. 6, Fig. 7, respectively. Increasing lecithin concentrations indicated a fast decrease of the color stability during storage. Moreover, the lower the extract and higher the lecithin concentration the faster the bleaching effect is apparent which is linked to the degradation of flavylium cation (Table 1).

**Table 1:** Symbols used in the study.

**Symbol**	**Meaning**
LE	Lecithin
E	Black Carrot Extract
_gel	Liposomes gel-filtrated
TEAC	Trolox Equivalent Antioxidant Capacity
L*	Lightness
a*	Redness
b*	Yellowness
Conc.	Concentration

## 2 Experimental design, materials and methods

Black carrot extract (BCE) (HF# 2.10592) (10 mg/g expressed as cyanidin 3-O-glucoside chloride) was provided by Döhler GmbH (AG Darmstadt, Germany). The soy lecithin (Lipoid S75) containing 69.3% phosphatidylcholine, 9.8% phosphatidylethanolamine, and 2.1% lysophosphatidylcholine was obtained from Lipoid AG (Ludwigshafen, Germany). Both were stored at −20 °C before the homogenization of liposomes.

Liposomes were generated via two step homogenization as described [2]. Sephadex gel filtration were performed to remove unencapsulated extract from liposomal samples [3] before analyses. Analyses of total phenolic compounds, anthocyanin content, and total antioxidant capacity were performed to both liposomes and gel filtered liposomes. The Folin-Ciocalteu reagent was used in total phenolic content analysis of the samples using the modified method [4] as described [5]. The anthocyanin content in the liposome samples was measured spectrophotometrically at 530 nm using the modified method [6] as described [7]. Color attributes (L*, a*, and b* values) of the liposomal samples were measured with a CR-400 handheld chroma meter (Minolta, Tokyo, Japan) during storage.

The free and extract loaded liposomes were stored at ambient temperature in the dark for 21 days. All the samples were stored in airtight containers as full volume. All the analyses were performed on the stored samples during the storage period.

All the experiments were performed at least 3 times for each triplicate sample. The average and standard deviation of all data were calculated using Microsoft Excel for Mac (version 15). Statistical analyses were performed using an one-way analysis of variance (ANOVA), followed by the Duncan post hoc test using SPSS software (version 21.0, SPSS, Chicago, IL, USA).
