# Supplementary material for: Additional data on stability of black carrot extract-loaded liposomes during storage
Source: Data Brief. 2018 Oct 9;21:562–7. doi: 10.1016/j.dib.2018.10.011 (PMC6199780; doi:10.1016/j.dib.2018.10.011)
Supplement: Supplementary file 1 — Supplementary material [file mmc1.docx]

Conflicts of Interest

We wish to confirm that there are no known conflicts of interest associated with this publication and there has been no significant financial support for this work that could have influenced its outcome.


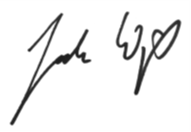


Prof. Dr. Jochen Weiss

Corresponding author
